# Long-term outcomes in recurrent diverticulitis: a systematic review of treatment strategies and recurrence rates

**DOI:** 10.1007/s00384-026-05196-6

**Published:** 2026-09-11

**Authors:** Kaiser O’Sahil Sadiq, Rishab Belavadi, Swetha Lakshminarayanan, Andres Fontaine-Nicola, Robert D. Bennett

**Affiliations:** 1 Department of Surgery, UC San Diego, 3855 Health Sciences Dr, San Diego, CA 92037 USA; 2 Department of Surgery, Mayo Clinic Arizona, Phoenix, AZ USA; 3 Division of Colon & Rectal Surgery, University of South Florida Morsani College of Medicine, Tampa, FL USA; 4 Department of Surgery, North Shore University Hospital, Zucker School of Medicine at Hofstra/Northwell, Manhasset, NY 11030 USA

**Keywords:** Outcomes, Recurrence, Quality of life, Diverticulitis, Surgery

## Abstract

**Background:**

Diverticulitis prevalence is on the rise, with recurrences occurring in up to one-third following an uncomplicated initial episode. While sigmoid resection was historically routine after a second episode, current guidelines recommend individualization. This review evaluates long-term outcomes for recurrent diverticulitis, comparing different treatment approaches.

**Methods:**

A systematic review (PROSPERO ID CRD42024600674) adhering to PRISMA guidelines searched several electronic databases. Clinical trials and observational studies published in English over the past decade that reported outcomes for persistent or recurrent diverticulitis were sought.

**Results:**

From 189 references, three retrospective cohort studies and one randomized control trial (RCT) were included, totaling 446 patients (42.1% male). Recurrence rates were 5.6–21.1% over follow-up durations ranging between 17 and 190 months. Definitions for recurrent diverticulitis varied across studies, with employed treatments including elective resection and nonoperative management. Outcomes evaluated included quality of life (QoL) assessments, reintervention rates, and recurrence risk. QoL showed greater improvement with surgery, but reintervention was more common after surgery (21%) than with nonoperative management. Recurrent or persistent diverticulitis demonstrated a higher recurrence risk, with increased incidence of surgery and comparable incidence of ostomy.

**Conclusions:**

Persistent or recurrent diverticulitis appears to be associated with higher rates of recurrence and surgery. However, contemporary literature detailing long-term outcomes is limited. Although surgical intervention may enhance QoL, it may also necessitate more reinterventions. Standardizing recurrent diverticulitis definitions would enable comparative analyses and inform guideline updates, facilitating evidence-based clinical decision-making.

## Introduction

The etiopathogenesis ofdiverticular disease remains to be conclusively determined; however, various mechanisms have been proposed to explain the development of diverticulosis, including insufficient dietary fiber intake, colonic dysmotility, and structural abnormalities of the colonic wall [1]. Diverticulitis, traditionally believed to result from the obstruction of colonic outpouchings leading to ischemia, microperforation, and subsequent infection [2], is now also thought to involve genetic predisposition, chronic inflammation, and alterations in gut microflora as contributing factors [3–9]. The incidence of diverticulitis has been on the rise [1], with recurrences occurring in up to one-third following an uncomplicated initial episode [10]. Each year, diverticular disease accounts for 200,000 hospitalizations and 371,000 visits to the emergency department, generating annual healthcare costs exceeding 2 billion USD [11, 12]. Historically, sigmoid resection was routinely indicated following an uncomplicated recurrence [1]; however, contemporary guidelines recommend individualization [13, 14]. This systematic review evaluates long-term outcomes for patients with recurrent diverticulitis, comparing operative and nonoperative management strategies. A narrative review outlining the management strategies and considerations has been included as part of the discussion.

## Methods

### Study design

This systematic review was conducted in accordance with PRISMA guidelines [15]. The study protocol was registered on PROSPERO with the ID CRD42024600674 [16]. The primary objective was to determine the long-term outcomes of recurrent diverticulitis in adults with at least two episodes of diverticulitis. The primary outcomes were the rate of recurrence, complications, reintervention, permanent stoma incidence, and quality of life metrics. Secondary outcomes were comparisons of outcomes between those who underwent surgical and nonsurgical management. Two independent reviewers assessed and appraised the quality of the included studies, followed by data extraction using a standardized table. Discrepancies between reviewers were resolved through discussion, and when necessary, a third reviewer was consulted to achieve consensus. Agreement statistics were not calculated.

### Search strategy

A comprehensive search of the literature published up to October 12, 2024, was conducted using the Cochrane Library, EMBASE, PubMed, PubMed Central, and Medline databases. The search string used was: (diverticulitis AND (recurrent OR recurrence OR persistent OR persistence OR ongoing) AND 'long-term outcomes'). No MeSH terms were used. Additional manual searches were conducted via Google Scholar and the reference lists of relevant articles.

### Inclusion and exclusion criteria

Eligible studies included clinical trials and cohort studies published in English within the past decade. Single-arm studies were included in addition to comparative studies. Exclusion criteria included other study designs (e.g., animal studies, in vitro studies, case reports, case series, case-control studies, conference abstracts), irretrievable studies, studies published in languages other than English, and those published more than 10 years ago. Studies lacking long-term outcomes (follow-up < 2 years) for uncomplicated recurrent or persistent diverticulitis were also excluded. Given that the American Society of Colorectal Surgeons (ASCRS) guidelines published in 2014 recommended an individualized approach to the management of acute diverticulitis, the authors of the present study elected to focus on contemporary evidence spanning a similar timeframe.

### Quality assessment

The quality of cohort studies was assessed using the Newcastle–Ottawa Scale (NOS) [17], with thresholds defined by the Agency for Healthcare Research and Quality (AHRQ) [18]. Randomized controlled trials (RCTs) were evaluated using the revised Cochrane Risk of Bias Tool (RoB 2) [19].

### Extracted data and variables

The extracted data included sample size, average age, gender distribution, follow-up duration, definitions of recurrence or persistence, treatment strategies, measured outcomes, and recurrence rates.

## Results

**Fig. 1:**
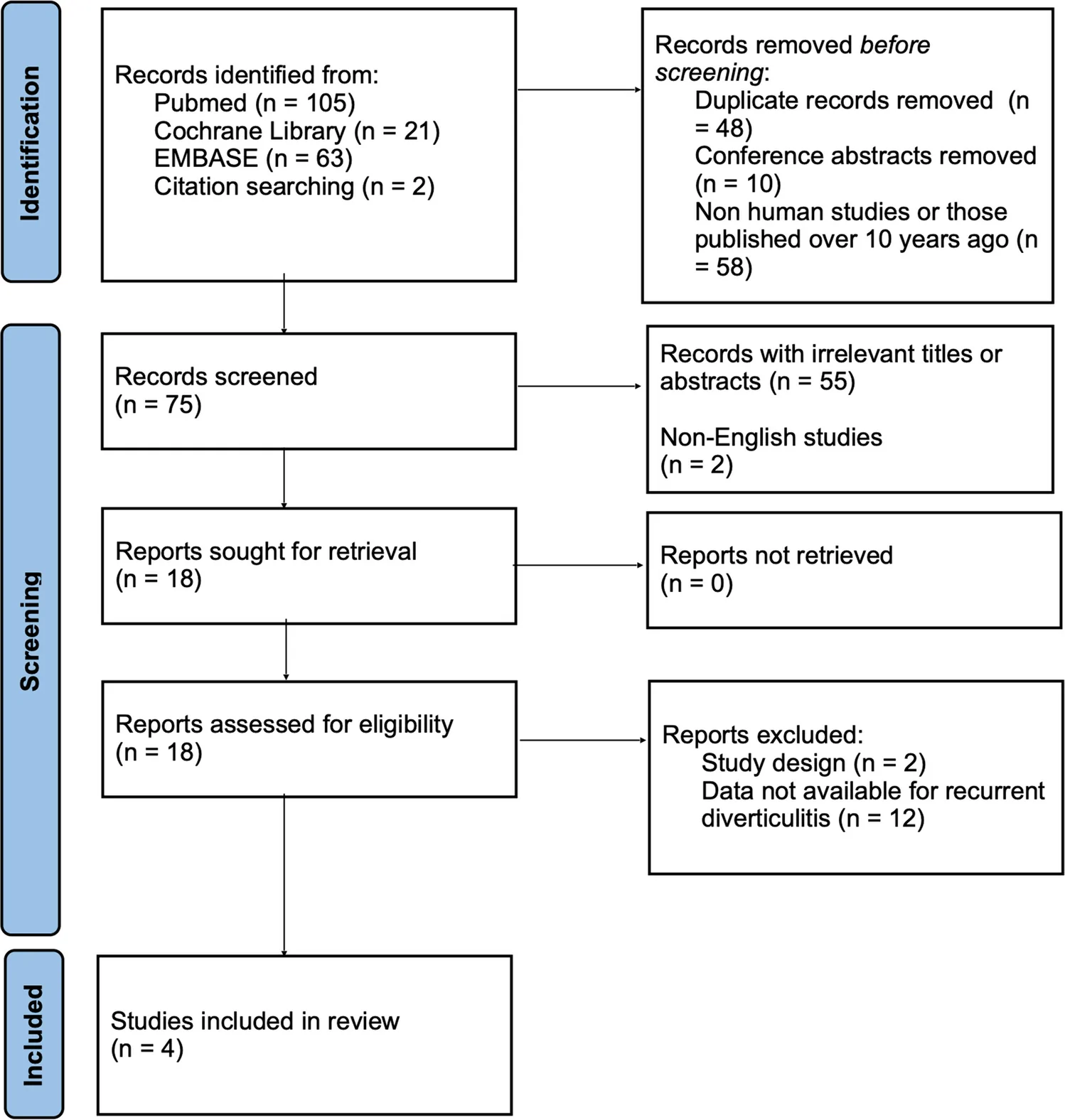
PRISMA (Preferred Reporting Items for Systematic Reviews and Meta-Analyses) flowchart of study selection

**Fig. 2:**
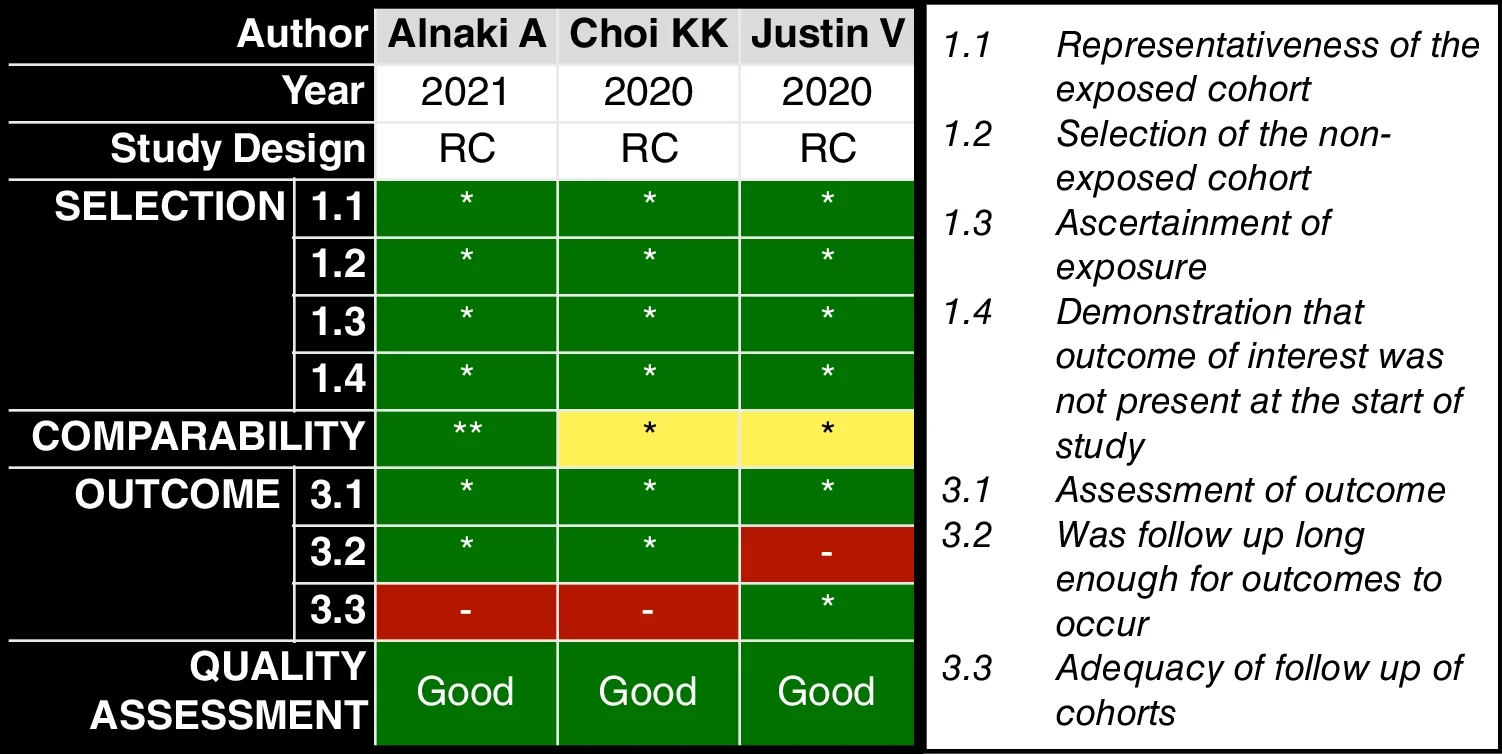
Chart depicting the quality of included cohort and case–control studies critically appraised using the Newcastle–Ottawa Scale and Agency for Healthcare Research and Quality thresholds. Abbreviations: *RC*, retrospective cohort

**Fig. 3:**
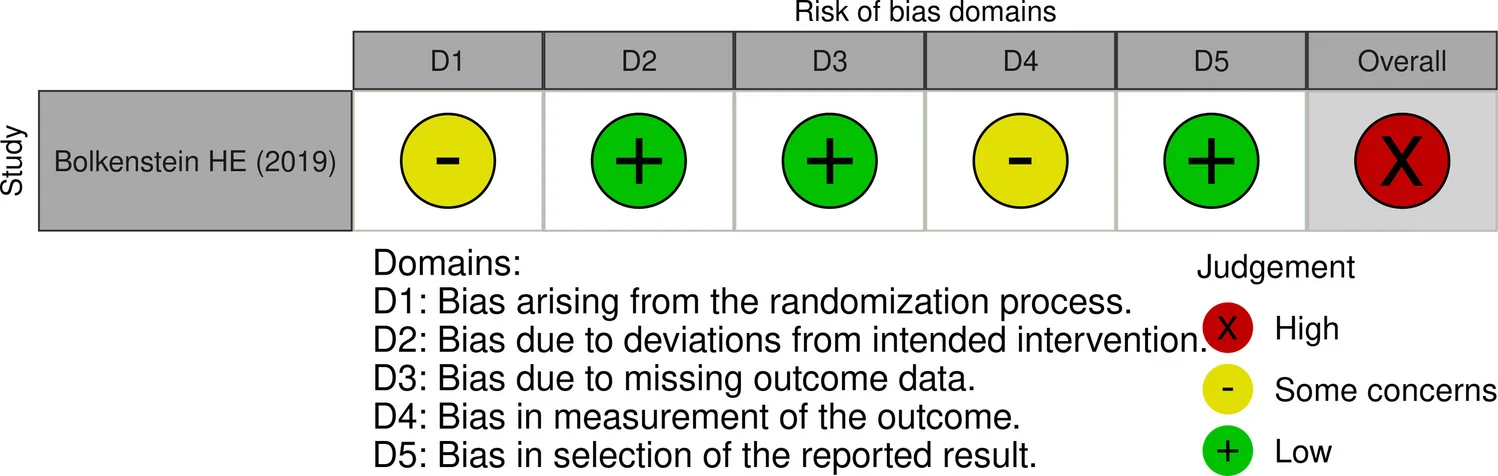
Traffic-light plot depicting the quality of included RCTs critically appraised using the RoB2 tool. Abbreviations: *RCT*, randomized controlled trial; *RoB2*, a revised tool for assessing risk of bias in randomized trials

The search strategy identified 189 references, with an additional 11 references retrieved from manual searches through Google Scholar and reference lists. After excluding 196 articles for various reasons (Fig. 1), the remaining studies underwent quality assessment as shown in Figs. 2 and 3. The final review included one RCT and three retrospective cohort studies. All cohort studies were determined to be of good methodological quality based on NOS and AHRQ thresholds [20–22]. However, two of the three studies did not control for all confounders [20, 21]. The follow-up period was not deemed adequate in one of the studies [20], and proportion of those who completed follow up was inadequate in the remaining two studies [21, 22]. The RCT was found to have a high risk of bias, with some concerns in domains related to randomization and outcome measurement [23]. Given the limited number of included studies, quantitative synthesis was not possible, and comparisons are qualitative in nature and based on low-certainty data.

### Study characteristics

The review included four studies, with extracted data summarized in Table 1. The total sample size was 446 patients (42.1% male) with a median of 94.5 (range, 75–182). The duration of follow-up ranged from 17 to 190 months across the three studies reporting this information [21–23]. Recurrent diverticulitis was inconsistently defined across studies; with studies defining recurrent diverticulitis as two or more episodes [20], two or more episodes within 60 days [22], two or more episodes within 2 years and/or ongoing symptoms for over three months [23], and abdominal pain in a patient with CT-confirmed diverticulitis [21]. Treatment strategies included elective resection and nonoperative management. Reported outcomes included quality of life (QoL) scores, reintervention rates, and recurrence risk. Persistent symptoms were not reported as an outcome in any of the included studies.

**Table 1:** Study characteristics and measured outcomes

Author (year)	Alnaki A (2021)	Bolkenstein HE (2019)	Choi KK (2020)	Justin V (2020)
Study design	Retrospective cohort	RCT	Retrospective Cohort	Retrospective Cohort
Sample size	75	109: 53 (S)/56 (C)	182	80: 36 (S)/44 (C)
Average age	Median (IQR) 57.0 (47.5–70.0)	Median (IQR) • S: 58.0 (50.0–67.0) • C: 60.5 (53.3–68.8)	Mean 56.2	NA
M:F	31:44	39:70	75:103	41:39
Follow-up duration (mo)	Median (range): 39.0 (17.0–67.3)	Mean: 60	Mean (range): 86 (6–190)	NA
Definition of recurrence/persistence	> 2 episodes within 60d	> 2 episodes within 2y and/or ongoing complaints (> 3 mo)	Abdominal pain episode with CT–confirmed diverticulitis	> 2 episodes
Treatment	NR	C: Lifestyle measures, dietary fiber, analgesics, or laxatives. S: Primary anastomosis (± DLI)	Elective laparoscopic sigmoid colectomy	Antibiotics, analgesics, bowel rest ± elective resection
Measured outcomes	Hazard ratios (95% CI) •Recurrence: 1.94 (1.37–2.76) •S: 5.11 (2.96–8.83) •Stoma: no significant difference	S/C (*p* < 0.05)—Mean ± SD •GIQLI: 118.2 ± 21.0/108.5 ± 20.0 •SF-36 physical: 47.6 ± 9.9/42.6 ± 10.5 •SF-36 Mental: 50.7 ± 9.4/46.0 ± 9.2 •VAS: 28.6 ± 27.9/44.1 ± 27.0 •EQ-5D: 0.85 ± 0.18/0.74 ± 0.21 -Reinterventions: 21%/4%	Univariate analysis- Increased recurrence rate (p = 0.049)	Mean ± SD: S/C •GIQLI: 91.70 ± 14.22/94.19 ± 15.33 (p = 0.36) •FIPS: 1.79 ± 0.64/NA •Complications: 5 (13.9%)/NA •Median time since last episode (range): 36 (2–141) mo/19 (1–55) mo (*p* < 0.001)
Recurrence (*n*)	NR	23 S: 6 (4 post surgery) C: 17	11	2 S: 2 C: NA

Abbreviations: *C*, conservative management; *d*, days; *EQ-5D*, EuroQol-5D; FIPS, Freiburger index for patient satisfaction; *GIQLI*, gastrointestinal quality of life questionnaire; *mo*, months; *NA*, not available; *NR*, not reported; *RCT*, randomized control trial; *S*, surgery; *SF-36*, Short Form 36; *VAS*, visual analog scale; *y*, years

### Recurrence

Recurrence rates were reported in three studies, ranging from 5.6% to 21.1% [20, 21, 23]. Two studies found a higher risk of recurrence in patients treated for persistent or recurrent diverticulitis, with an HR of 1.94 [95% CI, 1.37–2.76] [22] and a statistically significant association in univariate analysis (*p*= 0.049) [21], respectively. These studies did not compare treatment modalities. In Justin et al.'s retrospective study, the recurrence rate following surgery was 5.6%; however, recurrence rates in nonoperatively managed patients were unavailable as they were not followed up. [20] In the DIRECT trial, recurrence rates were 11% for surgically treated patients and 30% for those managed nonoperatively [23]. This corresponds to an odds ratio (OR) for recurrence of 0.29 [95% CI, 0.11–0.82] (*p* = 0.02) when comparing operative to nonoperative management.

### Quality of life

**Fig. 4:**
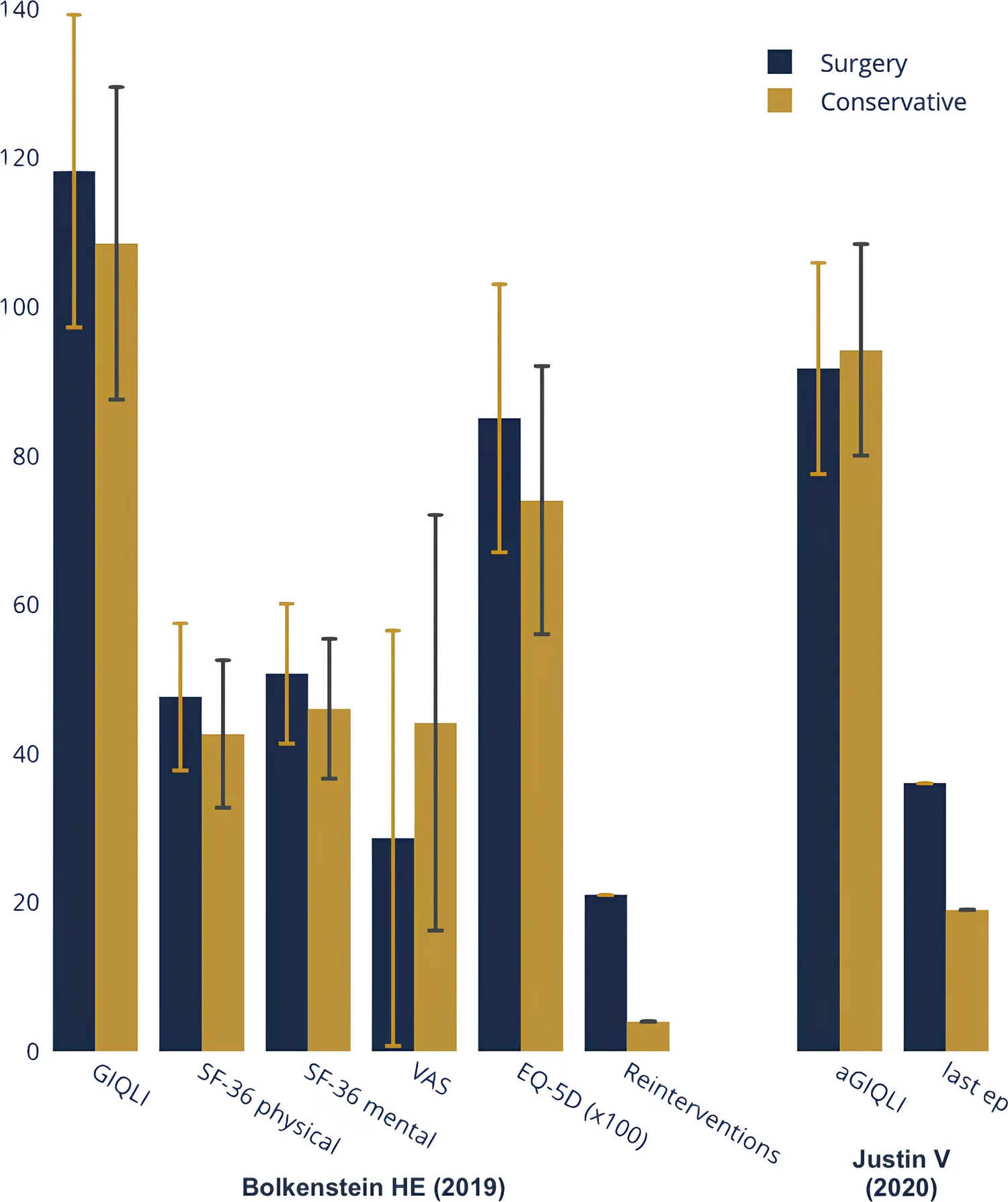
Graphical representation of studies comparing surgery to nonoperative management for recurrent diverticulitis. EQ-5D scores have been multiplied by 100 × to enhance visualization in relation to other QoL scores. Abbreviations: *EQ*-*5D*, EuroQol-5D; GIQLI, Gastrointestinal quality of life questionnaire; SF-36, Short Form 36; *VAS*, visual analog scale

Two studies assessed QoL outcomes, as illustrated in Fig. 4. Using the Gastrointestinal QoL Questionnaire (GIQLI), the RCT demonstrated significantly improved QoL following surgery at 5 years (118.2 vs 108.5, *p*= 0.02) [23], while a retrospective study reported a nonsignificant reduction in mean QoL scores (91.7 vs 94.2, *p*= 0.36) [20]. In the RCT, additional significant improvements were observed in Short Form 36 (SF-36) physical (47.6 vs 42.6, *p* = 0.03) and mental scores (50.7 vs 46.0, *p* = 0.01), EuroQol-5D (EQ-5D) score (0.85 vs 0.74, *p* = 0.02), and pain measured with a visual analog scale (28.6 vs 44.1, *p*= 0.01) [23]. The retrospective study also reported a longer duration from the last episode of diverticulitis in the surgical group (36 vs 19 months, *p*< 0.001), with high patient satisfaction (Freiburger Index of Patient Satisfaction score: 1.79) [20].

### Other outcomes

One study reported a higher incidence of surgery for persistent diverticulitis, with an HR of 5.11 [95% CI, 2.96–8.83], along with a comparable ostomy incidence [22]. The retrospective study by Justin found a complication rate of 13.9% in surgically treated patients [20]. The RCT reported increased reintervention rates for surgical management (21%) compared to nonoperative management (4%) [23].

## Discussion

Diverticulitis incidence has been on the rise [1], with a notable increase in younger individuals [24]. This shift has been attributed primarily to the rising rates of obesity, which is a known risk factor for diverticulitis [25]. Although the exact etiopathogenesis of diverticular disease remains elusive, several mechanisms have been proposed, including insufficient dietary fiber, dysmotility, and structural abnormalities of the colonic wall [1]. Diverticulitis, traditionally believed to result from the obstruction of colonic outpouchings leading to ischemia, micro-perforation, and subsequent infection [2], is now also thought to involve genetic predisposition, chronic inflammation, and alterations in gut microflora as contributing factors [3–9]. In the United States, diverticular disease is responsible for roughly 200,000 hospitalizations and 371,000 visits to the emergency department annually, resulting in healthcare expenditures exceeding 2 billion USD [11, 12]. Ethnic disparities have also been observed, with Caucasians comprising up to 70% of diverticulitis admissions [26], and women experiencing higher hospitalization rates [24, 26].

### Treatment guidelines

Over the past six decades, diverticulitis management has evolved significantly. Historically, elective sigmoidectomy was recommended following the initial episode in individuals younger than 50 and following the second episode in older patients [1]. This approach was predicated on the belief that elective resection would prevent recurrence and reduce the risk of emergent surgical intervention, and that younger patients experienced more virulent disease [1]. These recommendations were informed by 1960 s data showing that mortality from a second episode of diverticulitis was greater than the first by a factor of 2.5, with over 25% of cases requiring surgery [27]. Improved diagnostic imaging has since demonstrated that nonoperative treatment is feasible, even in cases of perforated diverticulitis with abscess formation [28]. Since 90% of diverticulitis cases are uncomplicated, nonoperative management is largely successful [1]. Recent studies have shown that the virulence of diverticulitis is similar across age groups, and younger patients only differ in having a longer lifespan during which recurrences can occur [1, 29]. Consequently, both the American Society of Colorectal Surgeons (ASCRS) and the World Society of Emergency Surgery now advocate for an individualized approach to management [13, 14]. The American Gastroenterology Association has also recommended an individualized approach, with nonoperative management being preferred [30]. The European Society of Coloproctology advises that decision-making should consider symptom severity, recurrence frequency, and patient comorbidities [31].

### Irritable bowel syndrome

There is growing interest in the potential overlap between diverticular disease and irritable bowel syndrome (IBS), with some suggesting that IBS shares pathophysiological mechanisms with diverticulitis [32–36]. It has also been proposed that diverticulitis may trigger a form of secondary IBS, similar to post-infectious IBS [37]. Some evidence even suggests that IBS may predispose individuals to recurrent episodes of diverticulitis [38]. However, the exact relationship between these conditions remains unclear, and whether they represent interconnected processes or distinct entities within a spectrum of disease is not well established [21]. Given that surgical intervention is not indicated for IBS, distinguishing between these conditions is crucial for appropriate management.

### Nonoperative management

High-fiber diets have been shown to reduce the risk of developing diverticulitis [1], and recommending a low-fiber or low-residue diet during acute episodes lacks scientific basis [39]. The previously held belief that seeds and nuts increase diverticulitis risk has been disproven [40], and avoiding these foods is no longer advised. Nonoperative management typically involves the use of inpatient antibiotics and bowel rest, but outpatient management may also be a viable option [1]. Recent evidence from the AVOD and DIABOLO trials suggests that antibiotics do not significantly affect the outcomes of diverticulitis, raising questions about their routine use[41, 42]. Mesalamine has been proposed as a treatment option given the overlap between diverticulitis and inflammatory bowel diseases; however, randomized controlled trials and a meta-analysis have demonstrated no significant benefit in using 5-ASA derivatives [43–45].

### Surgical resection

The standard surgical treatment for diverticulitis has long been the Hartmann’s procedure [1], but primary anastomosis is now preferred due to its association with improved restoration of intestinal continuity and shorter hospital stays at reversal [46, 47]. Resection should be limited to the thickened, inflamed, and noncompliant colonic segment, leaving otherwise healthy colon affected by diverticulosis intact [1]. Care should be taken to avoid including diverticula in the anastomosis, as this could increase the risk of dehiscence due to decreased structural integrity [1]. The risk of recurrence is significantly influenced by the location of the distal resection margin, with colosigmoid anastomosis associated with a fourfold increased risk of recurrence compared to colorectal anastomosis [48, 49]. In some cases, tension-free anastomosis may require mobilization of the splenic flexure or rectum [1]. Unlike oncologic resections, sigmoidectomy for diverticulitis does not require high ligation of the inferior mesenteric artery (IMA), and preserving the IMA has demonstrated better functional outcomes without increasing the risk of anastomotic leakage [50–52]. Although current evidence does not support routine IMA preservation [1], its benefits in select patients cannot be overlooked. As the prevalence of diverticulitis continues to rise alongside obesity rates, an increasing number of surgical cases will involve obese patients, who have greater postoperative complication risks [53, 54].

### Recurrence

Recurrence rates following nonoperative treatment range from 14 to 36% [10, 55–58], with complicated recurrences occurring in 3% to 5% of cases [10, 56, 58]. Most recurrences occur early, potentially indicating incomplete resolution of the initial episode [56]. Factors that may predict recurrence include a positive family history, involvement of more than 5 cm of the colon, and the presence of IBS [10]. Although the risk of recurrence is higher after a second episode [1, 59], and in younger patients [60], there is no increased risk of complicated recurrence [5, 10, 41, 60]. Surgical intervention reduces the likelihood of recurrence by 66% to 89% [61, 62], and our review found that surgery decreased the probability of recurrence by 71%, based on a single RCT with a high risk of bias, without a formal meta-analysis. After resection, the estimated risk of recurrence is 0.3%, 3.0%, and 6.3% at 1, 5, and 10 years, respectively [21]. Beyond 10 years, the recurrence risk remained stable through the end of the 15-year study period [21].

### Quality of life

Frequent recurrences and persistent symptoms can negatively impact quality of life (QoL) by 17% to 24% [63–65]. Although one study in our review did not find a significant QoL difference, an RCT with high-risk of bias reported QoL improvement in surgically treated patients using various assessment tools [23]. While it is possible that this improvement may be related to a placebo effect [66], the QoL of surgically treated patients has been shown to be comparable to that of healthy individuals [63–65]. Consistent with previous findings [65], a single study included in our review revealed that female patients experienced greater QoL improvements after surgery compared to their male counterparts [20], although other studies have not reported gender differences [67, 68]. Despite increased complications such as anastomotic leakage, protective stomas, and incisional hernias in the surgical arm of the DIRECT trial, surgery was associated with greater QoL improvements [23]. In the nonoperative arm, nearly half of the patients ultimately required sigmoidectomy, with lower baseline QoL being the only predictive factor for failure of nonoperative management [23]. Thus, early operative intervention should be considered in patients with significant QoL impairments. However, this is based on limited, low-certainty data.

## Limitations

Our review is limited by the paucity of available studies, inconsistent definitions of recurrent diverticulitis, and the heterogeneity of study designs. Our findings are based on low certainty data from small retrospective series, and a single high‑risk‑of‑bias RCT and should be interpreted cautiously. Long-term follow-up is inherently difficult and susceptible to nonresponse bias. Although RCTs are methodologically superior to observational studies, the sole RCT included in our review was found to have a high risk of bias. The ongoing Comparison of Surgery and Medicine on the Impact of Diverticulitis (COSMID) trial, a multi-center randomized superiority trial, aims to compare elective colectomy with nonoperative management for recurrent uncomplicated diverticulitis [69]. The early termination of the DIRECT trial due to recruitment challenges underscores the importance of the COSMID trial, which may yield more robust data to inform clinical decision-making. The search strategy employed may have resulted in missed studies and an expanded search using additional terminology may have yielded additional eligible studies.

## Conclusions

Persistent or recurrent diverticulitis appears to be associated with higher rates of recurrence and surgery. However, contemporary literature detailing long-term outcomes is limited. Although surgical intervention may enhance QoL, it may also necessitate more reinterventions. Standardizing recurrent diverticulitis definitions, stratified by frequency of recurrences, would enable comparative analyses and inform guideline updates, facilitating evidence-based clinical decision-making.

## Data Availability

No datasets were generated or analysed during the current study.

## Declarations

### Permission to reproduce material from other sources

NA.

### Disclaimer

Any opinions expressed in this paper are solely those held by the authors and are not representative of either the institution or the publisher.
